# Stable Isotope Labeling Highlights Enhanced Fatty Acid and Lipid Metabolism in Human Acute Myeloid Leukemia

**DOI:** 10.3390/ijms19113325

**Published:** 2018-10-25

**Authors:** Lucille Stuani, Fabien Riols, Pierre Millard, Marie Sabatier, Aurélie Batut, Estelle Saland, Fanny Viars, Laure Tonini, Sonia Zaghdoudi, Laetitia K. Linares, Jean-Charles Portais, Jean-Emmanuel Sarry, Justine Bertrand-Michel

**Affiliations:** 1 Centre de Recherches en Cancérologie de Toulouse, UMR1037, Inserm, Equipe Labellisée LIGUE 2018, F-31037 Toulouse, France; lucille.stuani@inserm.fr (L.S.); marie.sabatier@inserm.fr (M.S.); estelle.saland@inserm.fr (E.S.); sonia.zaghdoudi@inserm.fr (S.Z.); jean-emmanuel.sarry@inserm.fr (J.-E.S.); 2 Université de Toulouse, 31000 Toulouse, France; laure.tonini@inserm.fr (L.T.); jean-charles.portais@insa-toulouse.fr (J.-C.P.); 3 MetaToul-Lipidomic Core Facility, MetaboHUB, I2 MC, Inserm, 31100 Toulouse, France; fabien.riols@gmail.com (F.R.); aurelie.batut@inserm.fr (A.B.); viars.fanny1@gmail.com (F.V.); 4 LISBP, Université de Toulouse, CNRS, INRA, INSA, F-31077 Toulouse, France; pierre.millard@insa-toulouse.fr; 5 Centre de Recherches en Cancérologie de Toulouse, UMR1037, Inserm, Pôle Technologique, F-31037 Toulouse, France; 6 Institut de Recherche en Cancérologie de Montpellier, U1194 Inserm, Université de Montpellier, Equipe Labellisée LIGUE 2017, F-34090 Montpellier, France; laetitia.linares@inserm.fr; 7 MetaToul Core Facility, INSA, LISBP, F-31077 Toulouse, France

**Keywords:** lipidomics, isotopic profiling, metabolic reprogramming, IDH mutation, leukemia

## Abstract

**Background**: In Acute Myeloid Leukemia (AML), a complete response to chemotherapy is usually obtained after conventional chemotherapy but overall patient survival is poor due to highly frequent relapses. As opposed to chronic myeloid leukemia, B lymphoma or multiple myeloma, AML is one of the rare malignant hemopathies the therapy of which has not significantly improved during the past 30 years despite intense research efforts. One promising approach is to determine metabolic dependencies in AML cells. Moreover, two key metabolic enzymes, isocitrate dehydrogenases (IDH1/2), are mutated in more than 15% of AML patient, reinforcing the interest in studying metabolic reprogramming, in particular in this subgroup of patients. **Methods**: Using a multi-omics approach combining proteomics, lipidomics, and isotopic profiling of [U-^13^C] glucose and [U-^13^C] glutamine cultures with more classical biochemical analyses, we studied the impact of the IDH1 R132H mutation in AML cells on lipid biosynthesis. **Results**: Global proteomic and lipidomic approaches showed a dysregulation of lipid metabolism, especially an increase of phosphatidylinositol, sphingolipids (especially few species of ceramide, sphingosine, and sphinganine), free cholesterol and monounsaturated fatty acids in IDH1 mutant cells. Isotopic profiling of fatty acids revealed that higher lipid anabolism in IDH1 mutant cells corroborated with an increase in lipogenesis fluxes. **Conclusions**: This integrative approach was efficient to gain insight into metabolism and dynamics of lipid species in leukemic cells. Therefore, we have determined that lipid anabolism is strongly reprogrammed in IDH1 mutant AML cells with a crucial dysregulation of fatty acid metabolism and fluxes, both being mediated by 2-HG (2-Hydroxyglutarate) production.

## 1. Introduction

Cancer cells, including acute myeloid leukemia (AML) cells, grow and divide faster and more efficiently than normal cells, which increase their demand for energy, biosynthetic precursors, and macromolecular synthesis [1,2,3,4]. Most of them reprogram their metabolism from oxidative phosphorylation to aerobic glycolysis. The finding of this phenomenon, termed the “Warburg effect”, stimulated much research on tumorigenesis [4,5,6]. Over the past twenty years, advanced developments in genetic, omics and high-throughput screening methods have revealed that many of oncogenic signaling pathways regulate cell metabolism in cancer. Therefore, changes in cell metabolism represent a key hallmark in cancer biology [3] and it has been largely demonstrated that metabolic reprogramming in cancer cells occurs far beyond the Warburg Effect [7,8]. Indeed, cancer cells activate various metabolic pathways—e.g., glutaminolysis, amino acid degradation, fatty acid β-oxidation (FAO)—to generate the numerous precursors that are required for macromolecule biosynthesis, such as ribose for nucleic acids and glycerol for lipid synthesis. Dysregulation of lipid-associated pathways is increasingly described in tumors [9,10,11,12], and different studies have demonstrated that lipogenesis is significantly up-regulated in human cancers, in particular to respond to higher demands for membrane biogenesis [13,14,15] or to serve as energy source when nutrients are limited [16,17].

Few years ago, mutations in two key metabolic enzymes, isocitrate dehydrogenases (IDH1 and IDH2) have been discovered in gliomas and myeloid malignancies [18,19,20,21]. In AML, 15–20% of patients carry mutations in IDH1 or IDH2 [19,22,23,24,25]. This finding has reinforced the interest in studying cell metabolism in this pathology. IDH mutations induce a neomorphic activity resulting from a rearrangement of the enzyme active site favoring the reduction of α-ketoglutarate (α-KG) to d-2-hydroxyglutarate (2-HG) oncometabolite [26,27]. 2-HG strongly structurally resembles α-KG and can function as a potent competitive inhibitor of α-ketoglutarate-dependent enzyme reactions, including dehydrogenases, transaminases, and dioxygenases [28,29,30,31,32]. On the other side, the wild type enzyme catalyzes the interconversion between isocitrate and α-KG and produces NADPH, an essential cofactor that is required for numerous anabolic pathways (nucleotide, fatty acid elongation, lipid synthesis, and cholesterol synthesis) to sustain cell growth and proliferation [2], especially in cancer cells exhibiting aerobic glycolysis (Warburg phenotype), in hypoxia or with defective mitochondria [33,34,35]. IDH mutations are heterozygous with the conservation of a wild-type allele, suggesting the importance of this wild-type protein to favor the mutant activity. However, while the role of wild type IDH is well documented in normal and cancer cells, the impact of IDH mutation on lipid metabolism, and especially on its respective metabolic fluxes in cancer, is still largely unknown.

In this study we investigated lipid metabolism in AML cells harboring IDH1 R132H mutation, the most common IDH1 mutation. Lipids represent a very large class of molecules that show strong structural diversity (e.g., various combinations of fatty acyls and functional headgroups in phospholipids or various positions for hydroxyl groups on sterol). This chemical heterogeneity, together with the occurrence of many isomeric and isobaric lipid species and the large concentration range over which lipids are found, preclude the measurement of complete lipidomic profiles with a single analytical method. Specific methods are then used for each class of lipids: neutral lipids and fatty acids were analyzed by GC-FID, phospholipids and sphingolipids by LC-MS. Furthermore, the lack of analytical standards for complex lipids hampers the absolute quantification of many molecular species [36]. In this project, lipids were analyzed through different complementary approaches to get a broad coverage of the lipidome [37,38,39]. The data indicated significant changes in the lipidomic profile of IDH1 R132H cells as compared to WT cells, especially with the increase of phosphatidylinositol, ceramide, and monounsaturated fatty acid. These results encouraged us to investigate the dynamics of lipid synthesis in these cells. This was achieved by using ^13^C-labeling strategies in which the incorporation of ^13^C-label from [U-^13^C]-labeled glucose or [U-^13^C]-labeled glutamine into fatty acids was measured by GC-MS. The results showed increased de novo synthesis of fatty acids in IDH mutants through the production of 2-HG. Altogether, our investigations show that IDH1 mutation results in significant reprogramming of lipid metabolism in AML cells and could represent an interesting therapeutic target for this subgroup of patients.

## 2. Results and Discussion

### 2.1. Lipid Metabolism is Dysregulated in IDH1 Mutant Cells

**Figure 1:**
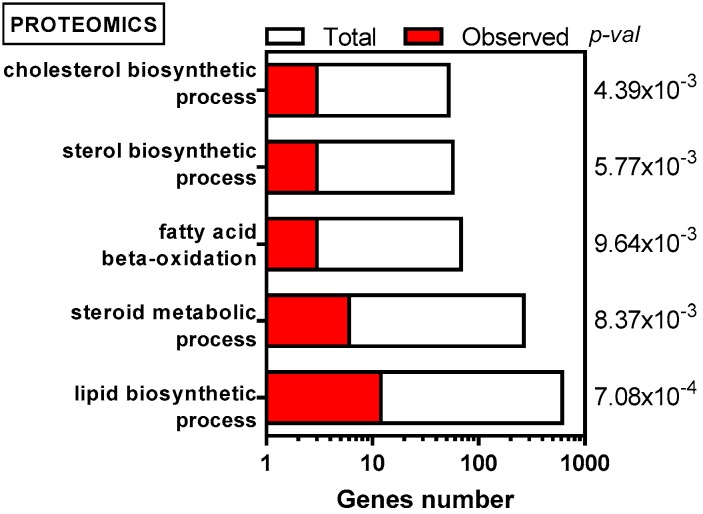
Enrichment in proteins associated with lipid metabolism in HL60 IDH1 R132H cells compared to WT cells (*n* = 4), based on GO biological processes. (Total) means all the genes encoding the proteins corresponding to the pathways described in the literature while (observed) refers to the genes encoding the proteins found more abundant in IDH1 R132H cells.

We first compared the proteome of IDH1 mutant HL60 cells to the one of IDH1 WT cells. A list of proteins that are significantly more abundant (fold change higher than 1.5 and FDR lower than 0.06) have been established (Supplementary Table S1B). Data mining of this specific protein set with Genomatix software revealed major changes in proteins that are associated to pathways of lipid biosynthesis and degradation (Figure 1), while proliferation rates remained unchanged and no significant differences in size, morphology, or doubling time for these cells have been observed. IDH1 R132H cells showed higher content in proteins that are involved in lipid synthesis, including cholesterol and sterol biosynthesis (IDI1, LSS, EBP; Supplementary Table S1). Interestingly, proteins involved in fatty acids (FA) oxidation were also significantly increased in IDH1 R132H cells (ACOX1/ACOX2, HSD17B4, Figure 1), suggesting higher FA catabolism to produce acetyl-CoA and feed the TCA cycle. FAO and lipogenesis are traditionally not described as being operating synchronously because they have opposite functions and are both regulated by ACC activity in opposite ways. However, some studies have demonstrated that FAO was essential to cell survival and metastasis in highly lipogenic solid cancers [40,41]. Of note, German and colleagues [42] have investigated this feature in AML cells. In fact, the authors demonstrated that under nutrient abundance, prolyl-hydroxylase 3 (PHD3) activates specifically ACC2 by hydroxylation, hence favoring malonyl-CoA formation and consequently inhibiting FAO. PHD3 does not act on ACC1, which could therefore maintain lipid synthesis while FAO is upregulated. Furthermore, 2-HG inhibition of α-KG-dependent dioxygenases, including PHDs, has been mainly described [28,43]. As a result, inhibition of PHD3 by 2-HG could prevent ACC2 hydroxylation and malonyl-CoA production to favor FAO in IDH1 mutant AML cells.

Moreover, we and others have shown that FAO and FA translocase/receptor CD36 played a crucial role in cell survival and drug resistance in AML in vitro and in vivo [44,45], thus reinforcing the key role of lipid metabolism in AML.

The comparison of the proteomes of IDH1 R132H and WT AML cells suggests a major reprogramming in the pathways of lipid degradation and biosynthesis, with potentially different impacts considering the diverse classes of lipids affected: mainly with sterol and fatty acids metabolism. A better understanding of this reprogramming process could give a precious insight into the biology of AML and the consequences of IDH1 mutation.

### 2.2. Lipidomic Experiments Confirmed Changes in Fatty Acid Reorganization in IDH1 R132H AML Cells

The lipidome of IDH1 R132H cells was measured using a combination of LC-MS and GC methods and compared to the one of WT cells. Lipids were extracted using a universal liquid-liquid extraction method [46] in the presence of internal standards (one per family studied) to follow the sample preparation and to perform relative quantification of the molecular species. Relative quantification of most lipids has been achieved by expressing the intensities of the peaks of interest relative to the area of the internal standard (Arbitrary Unit AU/million of cells). Absolute quantification (μg/million of cells) has been performed for LPC and sphingoïd bases due to the availability of pure standards. Phospholipids were measured by LC-MS/MS [37]. The different classes (PE, PC, PI, PS) were separated by polar head on a HILIC column (except for LysoPC, which were analyzed on an apolar column) [47]. Molecular species (with their number of carbons and number of double bonds) were discriminated by MS/MS based on specific MRM transitions.

**Figure 2:**
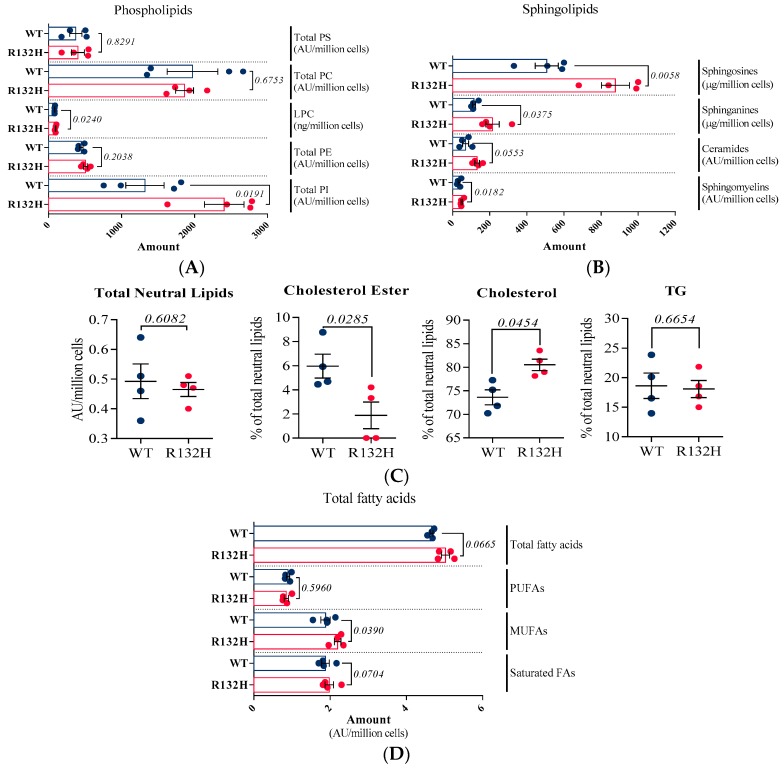
Lipidomic analysis in HL60 AML IDH1 WT (blue dots) and IDH1 R132H cells (red dots) (*n* = 4). (**A**) Phospholipids; (**B**) Sphingolipids; (**C**) Neutral lipids and percentages of each of its constituents; and, (**D**) Total Fatty Acids.

The relative amounts of PS, PE, and PC were similar in IDH1 WT and R132H cells (Figure 2A). This result is different from observations reported for gliomas harboring IDH mutation, suggesting a potential metabolic specificity of IDH mutation in AML cells. Indeed, Izquierdo-Garcia et al. [48] measured reduced PC levels while Reitman et al. [49] observed a decrease in PE levels in IDH1/2 mutant gliomas cells compared to IDH1 WT cells. More recently, Viswanath et al. [50] demonstrated that reduced PC and PE amounts in IDH mutant gliomas were due to a decrease in choline kinase and ethanolamine kinase, the enzymes that catalyze the production of PC and PE, respectively. However, we observed a significant increase for LysoPC (+15%) and mainly PI (+82%) families in AML mutant cells. The majority of the PI species were significantly increased, except 32:1; 34:2; 38:3; 38:4; 38:5; 40:5; and 40:6 (details of molecular species profiled for PI are listed in Supplementary Figure S1A). Modifications in intermediates of glycerophospholipid metabolism, such as LysoPC and PI, suggest that membrane trafficking and lipid signaling are stimulated in these cells [51].

Sphingolipids d18 (Cer and SM) were analyzed with the same method as phospholipids, while sphingoïds bases (sphinganine and sphingosine) were analyzed on a C8 column, and could be quantified due to appropriate standards [52]. Interestingly, the total amount of sphingolipids was increased by more than 40% in mutant cells and all of the four sphingolipids classes were enhanced (Figure 2B). For ceramides, if a global tendency to be increased in IDH1 mutant cells has been observed, significant changes have specifically been measured in *N*-(hexadecanoyl)-sphing-4-enine (Cer(d18:1/16:0)) and *N*-(docosanoyl)-sphing-4-enine (Cer(d18:1/22:0)) amounts (details of molecular species profiled for Cer are listed in Supplementary Figure S1B). Sphingomyelins are specific components of the cell membranes as they can form lipid rafts [53] that are essential for membrane protein dynamics and trafficking [51,54]. It is also well known that sphingolipids are key metabolites in oncogenic transformations [55].

Free and esterified cholesterol, as well as triacylglycerides (TG), were analyzed by GC-FID. The total amount of neutral lipids was unchanged between IDH1 WT and R132H cells, but the distribution of the molecular species was different. Indeed, we observed a decrease in esterified cholesterol in mutant cell counterbalanced by higher proportion of free cholesterol, while TG remained stable (Figure 2C).

Finally, the total FAs were profiled. Esterified FAs of the total extract (i.e., glycerolipids) were hydrolyzed in basic conditions and they were derivatized to be analyzed by GC-FID. A slight but significant increase in total FAs was observed in IDH1 mutant cells (+8%), which was mainly due to higher amounts of monounsaturated FAs (+17%). Polyunsaturated and saturated FAs remained stable (Figure 2D).

As expected regarding proteomics experiments, quantitative lipidomics data confirmed that IDH1 mutation leads to a re-organization of lipid metabolism, especially sphingolipids, lysoPC, the balance between cholesterol and cholesterol esters, and total FAs. In order to better understand how IDH1 mutation could be involved in the accumulation of lipids, we decided to apply stable isotope labeling experiments to identify pathways leading to total FA accumulation.

### 2.3. Isotopic Measurements of FAs Revealed Enhanced Lipid Anabolic Fluxes in IDH1 Mutant AML Cells

**Figure 3:**
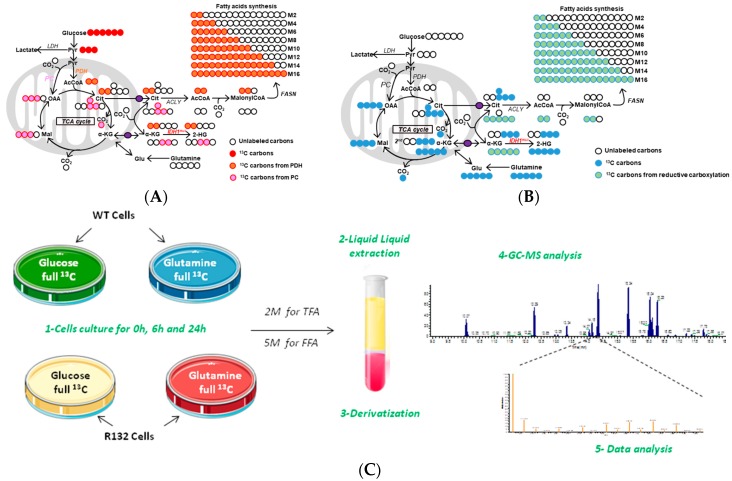
Simplified schematic of carbon atom (circles) transitions and tracers used to detect labeled fatty acids (FAs). (**A**) Isotopic label from [U-^13^C]glucose (red) to 2-HG and FAs synthesis through PC (Pyruvate Carboxylase; pink) or PDH (Pyruvate DeHydrogenase; orange); (**B**) Isotopic label from [U-^13^C]glutamine (blue) to 2-HG and FAs synthesis through classical TCA cycle (blue) or reductive glutamine metabolism (green). (**C**) Experimental design of the isotopic measurement of FAs on WT and IDH1 mutant cells.

Due to the size and structural diversity of lipids, methods that are based on stable isotopes are not so common to investigate the metabolism of FAs, glycerophospholipids, or sphingolipids species [Ecker, Progress in lipid research, 2014]. Current and main applications used labeled FAs to track metabolism of longer chain FA [56,57]. Here, to investigate the relationship between IDH1 mutation and FA production (Figure 3A,B), we investigated whether the mutation modified the conversion of the main nutrients that support cell proliferation, glucose, and glutamine, into FAs (Figure 3C). The conversion was measured from ^13^C-labeling experiments in which the incorporation of label into FAs from [U-^13^C]-labelled glucose or [U-^13^C]-labelled glutamine was measured by GC-MS. This approach provides quantitative information on the contribution of each carbon source to lipid biosynthesis [58] and further insight into specific pathways (e.g., reductive glutamine) by which the nutrient is converted into FA.

Analysis of FAs is commonly performed by GC-MS after derivatization of the carboxyl group of FAs with methyl ester (FAMEs) [38]. For FA quantification, the GC-MS is operated with electronic ionization, a high-energy ionization method that results in extensive fragmentation of molecules, and the molecular ion is too low to be detected [59]. This is detrimental for the purpose of isotopic profiling, for which the extent of ^13^C-atoms incorporation in FAs is derived from the isotopic cluster of the molecular ions. Hence, EI-GC-MS is not adapted to isotopic profiling of FAs. Indeed, our attempts to profile labelled FAs by classical methylation and EI-GC-MS were not conclusive. As an alternative, chemical ionization (CI) leads to much lower fragmentation, and thereby, better detection of (labeled) molecular ions than EI, though being slightly less robust [60]. We used a method in which FAs were derivatized with pentaflurobenzyl (PTF) and analysed by negative CI-GC-MS. Interestingly, the PTF group (*m*/*z* = 181 g/mol) is lost in the ionization source so that the major peak is a clear fragment corresponding to the molecular ion of the considered FA, providing high sensitivity. This major peak was followed by single ion monitoring (SIM) for each FA (Supplementary Table S2).

**Figure 4:**
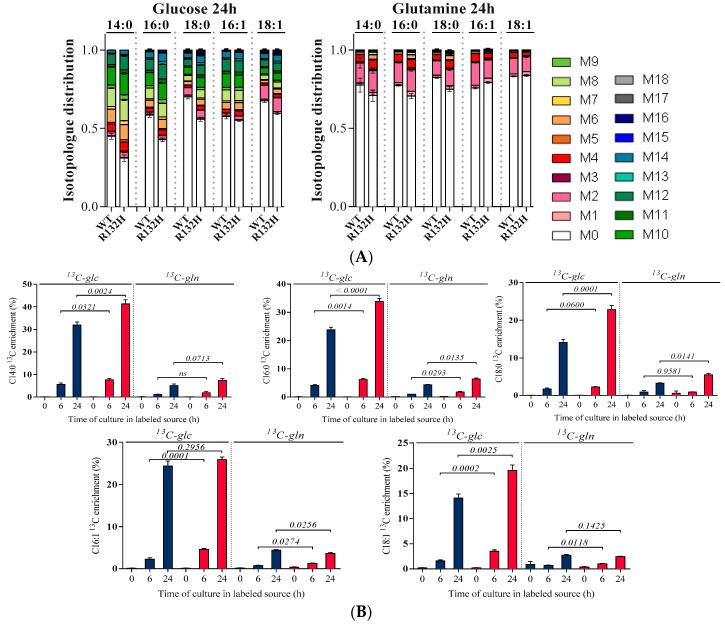
Isotopologues distribution (**A**) in C14:0; C16:0; C18:0; C16:1; and, C18:1 in HL60 IDH1 WT (in blue) and R132H (in red) at 24 h following and (**B**) ^13^C enrichment at 0, 6, or 24 h cultures on [U-^13^C]-glucose or [U-^13^C]-glutamine (*n* > 2).

IDH1 WT and R132H cells were grown in medium containing uniformly ^13^C-labeled glucose or ^13^C-labeled glutamine and dialyzed serum to avoid the dilution of label from traces of ^12^C-glucose or ^12^C-glutamine. To monitor incorporation of label into FAs, cells were sampled at different cultivation time points (0, 6, and 24 h). At each time-point, five millions of cells were collected and their lipids were extracted with a classical acidic extraction method adapted from the Bligh and Dyer protocol [46]. Extracted lipids were hydrolyzed with TFA (to hydrolyze esterified FFAs), derivatized with PTFBr after hydrolysis for TFA, and analysed by CI-GC-MS. The GC-MS profiles showed eight different FAs, with five of them (C14:0, C16:0, C16:1, C18:0, and C18:1) giving signals exploitable for isotopic profiling. For these FAs, the intensity of each isotopologue (M0, M + 1, M + 2, …, M + n) in the isotopic cluster of the molecular ion was measured (Table S2), and the distribution of carbon isotopologues (i.e., the fraction of molecules having incorporated 0, 1, 2, etc ^13^C atoms) was derived from these intensities after correction for ^13^C natural abundance using the sofware IsoCor [61]. Then, the molecular enrichment (average % of ^13^C-atoms in the molecule) was calculated (Figure 4).

For all conditions, we observed that the most abundant FA isotopologues contain an even number of ^13^C atoms, with very low fractions of isotopologues containing an odd number of ^13^C atoms (<0.03). These isotopic profiles are thus consistent with the known elongation mechanism of FAs by successive incorporation of C2 blocks from the acetyl moiety of AcCoA. For all FAs, molecular ^13^C-enrichments were higher on ^13^C-glucose than on ^13^C-glutamine, indicating that acetyl-CoA is mainly produced from glucose. From the enrichment data at 24h, it can be estimated that glucose contributed to FA biosynthesis 4–7 times more than glutamine. This ratio was similar in both cells, indicating no impact of IDH mutation on the contribution of the two carbon sources to FA biosynthesis. Importantly, the dynamics of ^13^C-incorporation was significantly faster for all FAs in R132H mutant cells as compared to WT cells. This was surprising since total FAs pools were increased in IDH1 R132H AML cells (Figure 2D), which was expected to result in lower relative label incorporation. The faster labeling dynamics in IDH1 R132H AML cells therefore revealed a significant increase in the rate of FA biosynthesis as compared to the WT cells, which resulted in an increased turnover of intermediates despite higher pools. This enhanced lipid anabolism in mutant cells demonstrates that the upregulation of the protein machinery for FA biosynthesis observed in R132H cells actually translates in terms of metabolic fluxes. While inferring absolute flux values from these data would require mathematical models of FA biosynthesis, these results demonstrated the applicability of the proposed workflow to infer flux information of lipid metabolism in mammalian cells.

### 2.4. Lipogenesis is Regulated by 2-HG Production in IDH1 Mutant Cells

**Figure 5:**
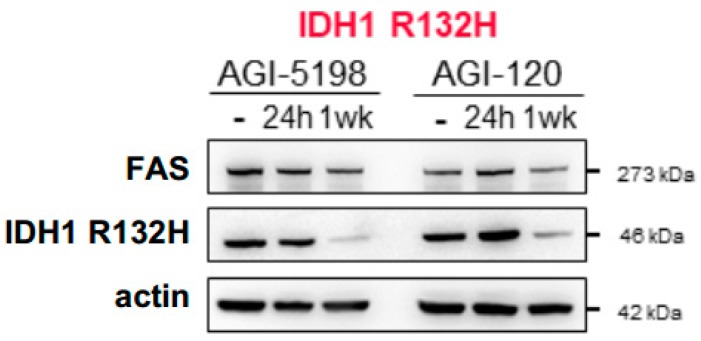
Fatty Acid Synthase (FAS) is linked to 2-HG production in IDH1 mutant cells. Lysates of IDH1 R132H AML cells in basal and following AGI treatments were immunoblotted with the indicated antibodies.

As all of the experiments described above showed that lipid biosynthesis is enhanced in IDH1 R132H cells, it was important to establish a more direct link between lipogenesis, 2-HG and IDH1 mutation. Therefore, we pharmacologically manipulated the amount of 2-HG using IDH1 mutation inhibitors AGI-5198 (the preclinical version of AG-120) and newly FDA approved AG-120 [62,63,64] during 24 h and one week for IDH1 R132H culture and we observed that the decrease in IDH1 R132H protein abundance correlated with the reduction in Fatty Acid Synthase (FAS) protein amount (Figure 5).

Mechanistically, several transcriptional factors, such as SREBP1/2, LXR, ChREBP, or CEBPα/β regulate de novo lipogenesis and lipid metabolism in various cell types. Interestingly, Ricoult et al. [65] have shown that, in two solid cancers, fibrosarcoma and colorectal carcinoma with IDH1 mutation, genetic invalidation of SREBP1/2 reduced 2-HG production. Moreover, knockdown of SREBP1/2 decreased FASN protein levels, mainly in fibrosarcoma. The differences observed into the two cell lines suggest that other transcription factors could regulate mutant IDH1 and lipogenesis depending on the oncogenic context. Notably, we have previously showed that IDH1 mutation and its oncometabolite (R)-2-HG induced an increase in CEBPα expression in epigenetic-dependent manner, and an activation to prime these cells to myeloid differentiation [66]. It would be of particular interest to study the regulation of lipid synthesis and IDH1 mutation by these different transcriptional factors in AML and to address, whereas IDH1 mutant inhibitors reverse this regulation to determine potential combinatory therapies.

Proteomic experiments on IDH1 mutant AML cells showed an upregulation of protein implicated in cholesterol and sterol biosynthesis and proteins that are involved in fatty acids oxidation. These modifications suggest a reprogramming in the pathways of lipid degradation and biosynthesis. It was then really interesting to characterize the lipidome of these cells versus the wild type one. The lipidomic approach used in this study showed an increase in phosphadidylinositol, ceramide, sphingosine and sphinganine, free cholesterol, and monounsaturated fatty acid (MUFA) species amounts and a decrease in cholesterol esters level in IDH1 mutant cells. In order to understand how IDH1 mutation could be involved in the increase of MUFA, we applied a stable isotope labeling experiments using ^13^C labeling after growing the cancer cells on uniformly ^13^C-labeled glutamine or labeled glucose. Dynamics of ^13^C-incorporation were clearly faster in IDH1 mutant cells. This enhanced lipid anabolism demonstrates that the upregulation of the protein machinery for FA biosynthesis observed in IDH1 mutant AML cells actually translates in terms of metabolic fluxes. Further questions will need to be investigated in order to evaluate the therapeutic possibilities of these findings. Is lipids’ dysregulation an Achilles’ heel of IDH1 mutant AML cells? Can we exploit it with specific inhibitors such as FASN inhibitors like C75 or orlistat? Are the lipid fluxes reversed by IDH1 mutant inhibitors or could the combination between lipolysis inhibitors and IDH1 mutant inhibitors lead to anti-leukemic effects? Our study highlighted the importance of lipids reprogramming in IDH1 mutant AML cells and paved the way for further studies that could lead to new therapeutic alternatives for this subgroup of AML patients.

## 3. Materials and Methods

### 3.1. Chemicals and Reagents

Acetonitrile (ACN) was HPLC-grade and purchased from Acros Organics (Geel, Belgium). Methanol HPLC-grade (MeOH), Dichloromethane (CH_2_Cl_2_), Ammonium Formate (>99%) (AF), Boron trifluoride-methanol solution 14% (BF3-MeOH), Heptane, Ethyl acetate (EtOAc), potassium hydroxide (KOH), pentafluorobenzyl bromide (PFB-Br), and diisopropylethylamine (DIPEA), iodoacetamide, ammonium bicarbonate, trifluoroacetic acid, and trypsin was supplied by Sigma Aldrich Chemicals Co. (Saint Quentin Fallavier, France), acetic acid (AA) from Honeywell Fluka. Ultrapure water (18.2 MΩ) was obtained from a milliQ apparatus from Millipore (Guyancourt, France).

Internal synthetic standards of phospholipids (PL: PE 12:0/12:0, PC 13:0/13:0, PS 12:0/12:0), Ceramides (Cer: Cer d18:1/15:0), sphingomyelins (SM: SM d18:1/12:0), and sphingosine (So: So d17:1) and sphinganine (Sa: Sa d17:0) were purchased from Avanti Polar Lipids (Alabaster, AL, USA). Synthetic internal standard PI 16:0/17:0 was supplied by J. Clark (Cambridge). Synthetic internal standards for neutral lipid (LN: stigmasterol, cholesteryl heptadecanoate, glyceryl trinonadecanoate) and for free FAs (FFA: heptadecanoate) and total FAs (TFA: glyceryl triheptadecanoate, glyceryl trinonadecanoate) were purchased from Sigma Aldrich (St Quentin Fallavier, France).

### 3.2. Cell Culture

Clones from the HL60 cell line expressing either IDH1 WT (#2, #4) or IDH1 R132H (#5, #11) were generated by our team [66]. These cell lines have been routinely tested for Mycoplasma contamination in the laboratory. They were maintained in minimum essential medium-α (MEMα, 22561-021, Gibco (Illkirch, France) supplemented with 10% FBS (Invitrogen, Villebon sur Yvette, France) in the presence of 100 U/mL of penicillin and 100 μg/mL of streptomycin (1% P/S), and were incubated at 37 °C with 5% CO_2_. The cultured cells were split every two to three days and maintained in an exponential growth phase. For starvations and ^13^C culture experiments, a specific MEMα media was ordered with the same formula than the one usually used in this paper (MEMα, 22561-021, Gibco), except that glucose, glutamate, glutamine, and pyruvate were removed. The media was first supplemented with 1% P/S and 5% dialyzed FBS (F0392, Thermo, Illkirch, France). Then, the media was supplemented with 5.6 mM ^12^C or ^13^C glucose, 1 mM ^12^C and 2 mM ^12^C or ^13^C glutamine.

### 3.3. Proteomics

#### 3.3.1. Protein Preparation

Three million cells of two independent experiments of two IDH1 WT (#2, #4) and two IDH1 R132H (#5, #11) clones (*n* = 4) were lysed using Tris buffer 50 mM pH 7.4, NaCl 150 mM and Chaps 1% during 15 min on ice. The lysates were then centrifuged 12,000 rpm, 15 min, 4 °C and the supernatants were collected. Proteins were first reduced using Laemmli Buffer (40 mM DTT final) at 95 °C during 5 min, then alkylated with iodoacetamide 90 mM for 30 min at RT in the dark. Next, protein migration was performed on 7.5% SDS PAGE and the gels were stained by Coomassie Blue. A unique band was cut and washed several times in ACN 100%, ammonium bicarbonate 100 mM and dried in vacuo. Gel pieces were rehydrated with 20 ng μL^−1^ trypsin prepared in ammonium bicarbonate 100 mM, and submitted to in gel-digestion overnight at 37 °C. Peptides were extracted and purified from gel and then subjected to mass spectrometry analysis.

#### 3.3.2. Analysis, Identification and Quantification of Proteins

Analysis of proteins was performed using a microLC system Ultimate 3000 (Dionex, Villebon sur Yvette, France) coupled to a Triple-TOF 5600+ (AB Sciex, Les Ullis, France) in the positive ion mode. Samples were first dissolved in 16 µL of buffer (5% ACN, 0.05% trifluoroacetic acid) and spiked with iRT calibration mix (Biognosys, Schlieren, France). The totality of the samples was then injected on a YMC-Pack Pro C18 column (3.0 mm × 150 mm; 3 µm particle size) at a flow rate of 5 µL.min^−1^. The run length was over 90 min with a gradient from 7% to 45% buffer B (buffer A: 0.1% formic acid, buffer B: 90% ACN, and 0.1% formic acid) in 70 min.

The MS data were acquired with a SWATH mode. The source parameters were set as follows: IS at 5500V, Cur gas at 25, GS1 at 5. The acquisition parameters were as follows: one 50 msec accumulation time MS scan followed by 50 variable SWATH windows each at 40 msec accumulation time for *m*/*z* 400–1235.

Identification was determined using an in-house SWATH library created from AML IDH1 WT and mutant cells with MaxQuant software, Les Ulis, France) (FDR 1%). A mass accuracy of 20 ppm on precursor ions was used, and 0.5 Da on the fragments. Cysteine carbamidomethylation, methionine oxidation, proline hydroxylation and serine, threonine and tyrosine phosphorylations were taken into account. Data treatment was done with Spectronaut Software 9.0, Les Ulis, France). First, relative abundance was calculated for each peptide (background noise normalized to 1) and the mean of the three most intense peptides for each protein were measured. Wilcoxon t-test was performed to determine differences between the two groups.

#### 3.3.3. Data Exploration and Mining

List of proteins (FC > 1.5 and FDR < 0.06) obtained throughout this study was uploaded in the Genome Analyzer bioinformatics tool (Genomatix, Les Ulis, France) for further functional analyses (GO term and small molecules) based on the Genomatix literature mining with a special interest in metabolic-linked pathways. The significance of the association between each list and functions or canonical pathways was measured by the Fisher’s exact test. As a result, a p-value was obtained, determining the probability that the association between the genes in our dataset and a function or canonical pathway can be explained by chance alone.

### 3.4. Lipidomic Analysis

#### 3.4.1. Preparation of Total Lipid Extracts

Cell pellet (of one million cells) was extracted adapted from Bligh and Dyer (B&D) [46] in CH_2_Cl_2_/MeOH with 2% AA/H_2_O (2.5: 2.5: 2, *v*/*v*/*v*), in the presence of the suitable internal standards. For PL, Cer, and SM relative quantification: 16 ng of Cer (d18:1/15:0), 180 ng of PE (12:0/12:0), 16 ng of PC (13:0/13:0), 16 ng of SM (d18:1/12:0), 30 ng of PI (16:0/17:0), and 156.25 ng of PS (12:0/12:0). For sphingoid bases: 5 ng of So (d17:1) and 5 ng of Sa (d17:0). For neutral lipid: 4 µg of stigmasterol, 4 µg of cholesteryl heptadecanoate, 8 µg of glyceryl trinonadecanoate, and for total FA: 2 µg of glyceryl triheptadecanoate.

#### 3.4.2. Phospholipid and Sphingolipid Relative Quantification

The lipid extract was dried, dissolved in 50 µL of MeOH then stored at −20 °C prior to analysis. Analysis were performed on an Agilent 1290 UPLC system coupled to a G6460 triple quadripole spectrometer (Agilent Technologies, Les Ulis, France)) using a Kinetex HILIC column (Phenomenex, Le Pecq, France), 50 × 4.6 mm, 2.6 µm). The column temperature was controlled at 40 °C. The mobile phases A and B were ACN and 10 mM AF in H_2_O at pH 3.2, respectively. The gradient was as follows: from 10% to 30% B in 10 min; 10–12 min, 100% B; and, then back to 10% B at 13 min for 1 min. The flow rate of mobile phase was 0.3 mL/min and the injection volume was 5 µL. Electrospray ionization (ESI) was employed at 325 °C in positive (for Cer, PE, PC, and SM analysis) and negative ion mode (for PI and PS analysis). The collision gas was nitrogen. Needle voltage was set at +4000 V. SRM transitions were used for relative quantification with a precursor ion scan of 184 *m*/*z*, 241 *m*/*z,* and 264 *m*/*z* to PC/SM, PI, and Cer, respectively; and, a neutral loss scan of 141 *m*/*z* and 87 *m*/*z* to PE and PS, respectively. In each family, individual molecular species were scanned with suitable SRM scan mode, the area of each peak were measured via Mass Hunter software. The relative quantitative calculations were based on the peak area ratios relative to the internal standards [37].

#### 3.4.3. Sphingoid Bases

The lipid extract was dried and dissolved in 50 µL of MeOH then stored at −20 °C prior to analysis. Analysis were performed on an Agilent 1290 UPLC system coupled to a G6460 triple quadripole spectrometer (Agilent Technologies, Les Ulis, France) an Acquity UPLC BEH-C8 (Waters, Issy les Moulineaux, France), 100 × 2.1 mm, 1.7 μm) maintained at 35 °C. The mobile phases A and B were H_2_O, FA (99.9:0.1; *v*/*v*), and ACN, FA (99.9:0.1, *v*/*v*), respectively. The gradient was as follows: 50% B at 0 min, 60% B at 2 min, 60% B at 3 min, 100% B at 4 min, 100% B at 8.5 min, and 50% B at 9 min. The flow rate of mobile phase was 0.3 mL/min and the injection volume was 5 µL. ESI was performed in positive ion mode at 300 °C. The collision gas was nitrogen. Needle voltage was set at + 4000 V. SRM transitions in neutral loss scan were used. For quantitative analysis, calibration samples (500 to 0.976 ng) were prepared with commercial sphingolipid standards. All of the quantitative calculations were based on the peak area ratios relative to the internal standards [adapted from Sikora [52].

#### 3.4.4. Neutral Lipid Relative Quantification

The lipid extract was dried, dissolved in 30 µL of EtOAc, and then stored at −20 °C prior to analysis. 1 µL of the lipid extract was analyzed by gas chromatography on a FOCUS Thermo Electron system using a Zebron-1 fused silica capillary column (Phenomenex, 5 m × 0.32 mm, 0.50 µm film thickness). Oven temperature was programmed from 200 °C to 350 °C at a rate of 5 °C/min and the carrier gas was hydrogen (0.5 bar). The injector and the detector temperatures were at 315 °C and 345 °C, respectively. All of the quantitative calculations were based on the chromatographic peak area relative to the internal standards [39].

#### 3.4.5. Total FA Profiling

The lipid extract was hydrolysed in KOH (0.5 M in MeOH) at 50 °C for 30 min, and transmethylated in 1 mL of BF_3_-MeOH and 1 mL of heptane at 80 °C for 1h. After the addition of 1 mL H_2_O to the crude, FAs methyl esters (FAMEs) extract was extracted with 3 mL of heptane, dried, and dissolved in 20 µL of EtOAc. 1 µL of FAMEs extract was analyzed by gas chromatography (GC) on a Clarus 600 Perkin Elmer system using a Famewax RESTEK fused silica capillary column (30 m × 0.32 mm, 0.25 µm film thickness). Oven temperature was programmed from 110 °C to 220 °C at a rate of 2 °C/min and the carrier gas was hydrogen (0.5 bar). The injector and the detector temperatures were at 225 °C and 245 °C, respectively. All of the quantitative calculations were based on the chromatographic peak area relative to the internal standards [38].

### 3.5. FA Isotopic Labeling Profiling

#### 3.5.1. Sample Preparation

Cell pellet (5 M) was extracted like previously in the presence of the internal standards: glyceryl trinonadecanoate (0.2 µg) for TFA and heptadecanoate (0.2 µg) for FFA. The equivalent of 4 M of cell was collected for the direct derivatization of FFA. Concerning TFA, the equivalent of 1 M of cells was hydrolysed in KOH (0.5 M in MeOH) at 50 °C for 30 min. Free and total FA were derivatized in pentafluorobenzyl esters with 1% PFB-Br and 1% DIPEA in ACN (50 μL), at RT for 20 min. Samples were dried and dissolved in EtOAc (10 µL).

#### 3.5.2. GC-MS Analysis

Labelled total FA analysis were performed on a Thermo Fisher Trace GC system that was connected to a ThermoFisher TSQ8000 (Les Ulis, France)) triple quadrupole detector using a HP-5 MS capillary column (30 m × 0.25 mm, 0.25 µm film thickness). Oven temperature was programmed, as follows: 150 °C for 1 min, 8 °C/min to 350 °C, then the temperature is kept constant for 2 min. The carrier gas was helium (0.8 mL/min). The injector, the transfer line, and the ion source temperature were at 250 °C, 330 °C, and 300 °C, respectively. The TSQ8000 was operated in negative ionization mode (Methane at 1 mL/min) in selected ion monitoring (SIM) mode and 1 µL of sample was injected in splitless mode.

#### 3.5.3. Data Processing

GC-MS analysis produced a mass spectrum for each FA, which contains the abundance of each isotopologue. For each FA, the lightest (unlabeled) isotopologue is denoted M + 0; e.g., PFB-palmitate M + 0 has a mass of 255.3, whereas the isotopologue with 1 atom [^13^C] PFB-palmitate (M + 1) has a mass of 256.3, etc., (Supplementary Table S1). Isotopic clusters were obtained by integrating gas chromatographic signals for each isotopologue. Isotopologue distributions were obtained from the corresponding isotopic clusters after correction for natural abundance of carbon and non-tracer elements (oxygen and hydrogen) using the software IsoCor, and purity of the tracer was corrected assuming 99% ^13^C-purity. Finally, the ^13^C-enrichment, which represents the mean content in tracer atoms (^13^C) within the molecule, was calculated from the corresponding IDs, as detailed in Millard et al. [61].

### 3.6. Immunoblotting of Total Proteins

For immunoblot assay, cells were first subjected to lysis in NuPAGE LDS Sample Buffer. Total proteins content from every samples was measure using Pierce BCA Protein Assay Kit according to the manufacturer’s recommendations. Samples lysates were then loaded onto NuPAGE 4–12% Bis-Tris Protein Gels (10, 12, or 15 wells). After electrophoresis, proteins were transferred to nitrocellulose membranes. The transblotted membranes were blocked for 1 h and then probed with appropriate primary antibodies (dilution as recommended by manufacturers) overnight at 4 °C. Next, the membranes were washed three times for a total of 30 min and then incubated with secondary antibodies at room temperature for 1 hr. After another three washes, proteins were detected using SuperSignal West Pico PLUS chemiluminescent Substrate, PXi imager (Syngene), and GeneSys software (Syngene, Paris, France), according to the manufacturer’s manual. Proteins expression were quantified using GeneTool software (Syngene) and normalized to their corresponding loading control. Antibodies for immunoblotting were purchased from the following sources: FASN (#3180S) from Cell Signaling Technology; Actin (MAB1501) from Millipore; IDH1 R132H (DIA-H09) from Dianova. HRP conjugate anti-rabbit (W4011) and anti-mouse (W4021) secondary antibodies were purchased from Promega (Charbonnières les Bains, France).

### 3.7. ChIP Assays

To perform chip assays briefly, 10^7^ cells were cross-linked in 1% formaldehyde/1% paraformaldehyde for 5 min, followed by addition of 125 mM Glycine to stop the reaction. Cells were then washed in PBS, resuspended in lysis buffer (10 mM Tris pH 8, 140 mM NaCl, 0.5 mM EGTA, 0.1% SDS, 0.5% Triton X-100, 0.05% NaDoc and protease inhibitors) and chromatin was shared by sonication. qChIPs were carried out by incubating cell lysates (Input) with 20 μL of protein G-Dynabeads and 5 ug of antibody. The same amount of rabbit IgGs (Santa Cruz, Boulogne Billancourt, France) was used for control ChIP experiments. After O/N incubation, washing, reverse cross-linking, and treatment with both RNase A and Proteinase K, proteins were removed with phenol/chloroform extraction and DNA was recovered using the NucleoSpin Extract II kit. Input and immunoprecipitated DNA were then analyzed by QPCR using the SYBR Green Master mix on a LightCycler 480 SW 1.5 apparatus (Roche, Boulogne Billancourt, France). Results are represented as the mean value of at least three independent experiments of immunoprecipitated chromatin (calculated as a percentage of the input) with the indicated antibodies after normalization by a control ChIP performed with rabbit IgGs.

### 3.8. Statistical Analysis

Statistical analyses were conducted using Prism software v6.0 (GraphPad Software, La Jolla, CA, USA). Statistical significance was determined by the two-tailed unpaired Student’s *t*-test. A *p*-value < 0.05 was considered to be statistically significant. The statistical parameters (i.e., exact value of *n*, *p*-values) have been noted in the figures and figure legends. Unless otherwise indicated, all data represent the mean ± standard error of the mean (SEM) from at least three independent experiments.

## 4. Conclusions

The combination of proteomics, lipidomics, and isotopic profiling experiments allowed us to uncover a profound reprogramming of lipid metabolism in IDH1 mutant AML cells through a simultaneous increase of both FA oxidation and de novo lipogenesis. This reprogramming is—at least partly- dependent on 2-HG production, which controls FAS expression. Integration of all of these omics data in an AML metabolic network could allow fluxes calculations to gain even more insight into the metabolic regulation of IDH WT and R132H cells.

## Supplementary Materials

The following are available online at http://www.mdpi.com/1422-0067/19/11/3325/s1.

## Abbreviations

AA	Acetic acid
ACN	Acetonitrile HPLC-grade
AML	Accute Myeloide Leukemia
AraC	Cytarabine
AU	Arbitrary Unit
B&D	Bligh and Dyer
BF_3_-MeOH	Boron trifluoride-methanol solution 14%
Cer	Ceramides
CH_2_Cl_2_	Dichloromethane HPLC-grade
ESI	Electrospray ionization
FA	Fatty acids
FAMEs	Fatty acid methyl esters
FAO	Fatty acids oxidation
FID	Flame Ionization Detector
GC	Gas chromatography
GO	Gene Ontology
H_2_O	Ultrapure water
HPLC	High performance liquid chromatography
IDH	Isocitrate dehydrogenase
2-HG	2-hydroxyglatarate
KOH	Acetic acid potassium hydroxide
LC-MS/MS	Liquid chromatography coupled to detector MS/MS
LOD	Limit of detection
LOQ	Limit of quantification
*m/z*	Mass-to-charge ratio
MeOH	Methanol HPLC-grade
MS	Mass spectrometry
PC	Phosphatidylcholine
PE	Phosphatidylethanolamine
PI	Phosphatidylinositol
PL	Glycerophospholipids
PS	Phosphatidylserine
PTFB-Br	Pentafluorobenzyl-bromide
PUFAs	Polyunsaturated fatty acids
RLCs	Resistant leukemic cells
S/N	Signal to noise ratio
Sa	Sphinganine
SIM	Selected ion monitoring
SM	Sphingomyelins
So	Sphingosine
SRM	Selected-reaction monitoring
MRM	Multiple-reaction monitoring
MS	Mass SPectrometry
UPLC	Ultra performance liquid chromatography
WT	Wild Type
