# Supplementary material for: Stable Isotope Labeling Highlights Enhanced Fatty Acid and Lipid Metabolism in Human Acute Myeloid Leukemia
Source: Int J Mol Sci. 2018 Oct 25;19(11):3325. doi: 10.3390/ijms19113325 (PMC6274868; doi:10.3390/ijms19113325)

Supplementary

Stable Isotope Labeling Highlights Enhanced Fatty Acid and Lipid Metabolism in Human Acute Myeloid Leukemia

Lucille STUANI[1,2], Fabien RIOLS[3], Pierre Millard[5,] Marie SABATIER[1,2], Aurélie BATUT[3], Estelle SALAND[1,2], Fanny VIARS[3], Laure Tonini[2,4], Sonia Zaghdoudi[1,2], Laetitia K. Linares[6], Jean-Charles PORTAIS[2,7], Jean-Emmanuel SARRY[1,2] and Justine BERTRAND-MICHEL[2,3,*]

Supplementary Table S1A. proteomics signature UP IDH R132H.

GO-Term	GO-Term id	P-value	# Genes (observed)	# Genes (expected)	# Genes (total)	List of observed genes
cholesterol biosynthetic process	GO:0006695	4,39E-03	3	0,330522766	49	IDI1, LSS, EBP
sterol biosynthetic process	GO:0016126	5,77E-03	3	0,364249578	54	IDI1, LSS, EBP
fatty acid beta-oxidation	GO:0006635	9,64E-03	3	0,438448567	65	ACOX2, HSD17B4, ACOX1
steroid metabolic process	GO:0008202	8,37E-03	6	1,753794266	260	IDI1, ACOX2, ACBD3, LSS, EBP, HSD17B4
lipid biosynthetic process	GO:0008610	7,08E-04	12	4,033726813	598	NANS, IDI1, ACOX2, ACBD3, IDH1, LSS, LTA4H, EBP, PCYT2, IMPA1, HSD17B4, CBR1

Supplementary Table S1B. List of protein UP IDH R132H.

	PG Gene	PG_ProteinDescriptions
PPP2R2A	PPP2R2D	Serine/threonine-protein phosphatase 2A 55 kDa regulatory subunit B alpha isoform,Serine/threonine-protein phosphatase 2A 55 kDa regulatory subunit B alpha isoform,Serine/threonine-protein phosphatase 2A 55 kDa regulatory subunit B alpha isoform,Serine/threonine-protein phosphatase 2A 55 kDa regulatory subunit B alpha isoform,Serine/threonine-protein phosphatase 2A 55 kDa regulatory subunit B alpha isoform;Serine/threonine-protein phosphatase 2A 55 kDa regulatory subunit B,Serine/threonine-protein phosphatase 2A 55 kDa regulatory subunit B,Serine/threonine-protein phosphatase 2A 55 kDa regulatory subunit B
HIBADH		3-hydroxyisobutyrate dehydrogenase, mitochondrial,3-hydroxyisobutyrate dehydrogenase, mitochondrial;3-hydroxyisobutyrate dehydrogenase
ACOX1	ACOX2	Acyl-coenzyme A oxidase
AAAS		Aladin,Aladin;Achalasia, adrenocortical insufficiency, alacrimia (Allgrove, triple-A) variant (Fragment)
ADD3		Gamma-adducin,Gamma-adducin;Adducin 3 (Gamma), isoform CRA_a;Adducin 3 isoform a variant (Fragment),Adducin 3 isoform a variant (Fragment)
AFG3L2		AFG3-like protein 2;AFG3 ATPase family gene 3-like 2 (Yeast), isoform CRA_a (Fragment);Similar to AFG3 ATPase family gene 3-like 2 (Yeast) (Fragment)
RNPEP		Aminopeptidase B
ARID1A		AT-rich interactive domain-containing protein 1A
ARID3A		AT-rich interactive domain-containing protein 3A
HEL-S-68p	HEL-S-272	Phosphoglycerate kinase
MROH7		cDNA FLJ58649;Maestro heat-like repeat-containing protein family member 7,Maestro heat-like repeat-containing protein family member 7,Maestro heat-like repeat-containing protein family member 7,Maestro heat-like repeat-containing protein family member 7,Maestro heat-like repeat-containing protein family member 7
NA		cDNA FLJ54032, highly similar to Elongation factor 1-alpha 1
BASP1		Brain acid soluble protein 1
BAZ1A		Bromodomain adjacent to zinc finger domain protein 1A;Bromodomain adjacent to zinc finger domain, 1A, isoform CRA_c
BOD1L1		Biorientation of chromosomes in cell division protein 1-like 1
BRD4		Bromodomain-containing protein 4;Bromodomain containing 4, isoform CRA_b
BROX		BRO1 domain-containing protein BROX,BRO1 domain-containing protein BROX;cDNA FLJ50123
C1orf234		Uncharacterized protein C1orf234
CA2		Carbonic anhydrase 2,Carbonic anhydrase 2,Carbonic anhydrase 2;Epididymis luminal protein 76
CAPN2		Calpain-2 catalytic subunit;cDNA FLJ39928 fis, clone SPLEN2021273, highly similar to Calpain-2 catalytic subunit (EC 3.4.22.53);Calpain 2, large [catalytic] subunit variant (Fragment);cDNA FLJ58224, highly similar to Calpain-2 catalytic subunit (EC 3.4.22.53);cDNA FLJ42761 fis, clone BRAWH3002574, highly similar to Calpain 2, large;cDNA, FLJ96158, highly similar to Homo sapiens calpain 2, (m/II) large subunit (CAPN2), mRNA;cDNA FLJ58517, highly similar to Calpain-2 catalytic subunit (EC 3.4.22.53)
CTSB		Cathepsin B,Cathepsin B;Cathepsin B, isoform CRA_a;cDNA FLJ78235;cDNA FLJ58073, moderately similar to Cathepsin B (EC 3.4.22.1);Cathepsin B (Fragment),Cathepsin B (Fragment),Cathepsin B (Fragment),Cathepsin B (Fragment),Cathepsin B (Fragment),Cathepsin B (Fragment),Cathepsin B (Fragment),Cathepsin B (Fragment),Cathepsin B (Fragment),Cathepsin B (Fragment),Cathepsin B (Fragment),Cathepsin B (Fragment),Cathepsin B (Fragment),Cathepsin B (Fragment),Cathepsin B (Fragment),Cathepsin B (Fragment),Cathepsin B (Fragment);cDNA FLJ59133, highly similar to Cathepsin B (EC 3.4.22.1);cDNA FLJ40065 fis, clone TESOP2000400, highly similar to CATHEPSIN B (EC 3.4.22.1)
CTSD	HEL-S-130P	Cathepsin D,Cathepsin D;Cathepsin D (Lysosomal aspartyl peptidase), isoform CRA_a;Cathepsin D (Fragment),Cathepsin D (Fragment),Cathepsin D (Fragment)
CTSZ		Cathepsin Z
CPD		Carboxypeptidase D
CBR1		Carbonyl reductase [NADPH] 1
CCAR1		Cell division cycle and apoptosis regulator protein 1
CHD1L		Chromodomain-helicase-DNA-binding protein 1-like,Chromodomain-helicase-DNA-binding protein 1-like,Chromodomain-helicase-DNA-binding protein 1-like,Chromodomain-helicase-

		DNA-binding protein 1-like,Chromodomain-helicase-DNA-binding protein 1-like,Chromodomain-helicase-DNA-binding protein 1-like;Chromodomain helicase DNA binding protein 1-like isoform A (Fragment)
CHD5		Chromodomain-helicase-DNA-binding protein 5
CLTCL1		Clathrin heavy chain 2
CNOT10		CCR4-NOT transcription complex subunit 10,CCR4-NOT transcription complex subunit 10;CCR4-NOT transcription complex subunit 10 (Fragment),CCR4-NOT transcription complex subunit 10 (Fragment),CCR4-NOT transcription complex subunit 10 (Fragment)
NaN		NaN
NaN		NaN
COPG1		Coatomer subunit gamma-1
COPG2		Coatomer subunit gamma-2
CAPNS1		Calpain small subunit 1,Calpain small subunit 1,Calpain small subunit 1,Calpain small subunit 1,Calpain small subunit 1;Calpain small subunit 1 (Fragment),Calpain small subunit 1 (Fragment),Calpain small subunit 1 (Fragment),Calpain small subunit 1 (Fragment),Calpain small subunit 1 (Fragment),Calpain small subunit 1 (Fragment),Calpain small subunit 1 (Fragment)
COPS8		COP9 signalosome complex subunit 8,COP9 signalosome complex subunit 8,COP9 signalosome complex subunit 8;COP9 constitutive photomorphogenic homolog subunit 8 (Arabidopsis), isoform CRA_a;cDNA, FLJ93976, highly similar to Homo sapiens COP9 homolog (COP9), mRNA;cDNA FLJ60469, highly similar to COP9 signalosome complex subunit 8
ITGB5	ITGB4; ITGB; ITGB2; ITGB6 ; ITGB3	Integrin beta
ASAH1		N-acylsphingosine amidohydrolase (Acid ceramidase) 1, isoform CRA_c;N-acylsphingosine amidohydrolase (Acid ceramidase) 1 preproprotein isoform a variant (Fragment);cDNA FLJ77858, highly similar to Homo sapiens N-acylsphingosine amidohydrolase (acid ceramidase) 1 (ASAH1), transcript variant 1, mRNA;cDNA FLJ40980 fis, clone UTERU2014464, highly similar to ACID CERAMIDASE (EC 3.5.1.23)
HSD17B4		Peroxisomal multifunctional enzyme type 2,Peroxisomal multifunctional enzyme type 2,Peroxisomal multifunctional enzyme type 2,Peroxisomal multifunctional enzyme type 2,Peroxisomal multifunctional enzyme type 2;cDNA, FLJ92803, highly similar to Homo sapiens hydroxysteroid (17-beta) dehydrogenase 4 (HSD17B4), mRNA
DNM1L		Dynamin-1-like protein,Dynamin-1-like protein,Dynamin-1-like protein,Dynamin-1-like protein,Dynamin-1-like protein,Dynamin-1-like protein;Dynamin 1-like, isoform CRA_f;Dynamin 1-like, isoform CRA_a;Dynamin 1-like, isoform CRA_c;cDNA FLJ56381, highly similar to Dynamin-1-like protein (EC 3.6.5.5)
EBP		3-beta-hydroxysteroid-Delta(8),Delta(7)-isomerase;Emopamil binding protein
HADHA		Trifunctional enzyme subunit alpha, mitochondrial;Epididymis tissue sperm binding protein Li 14m
EIF3M		Eukaryotic translation initiation factor 3 subunit M
ELMOD2		ELMO domain-containing protein 2,ELMO domain-containing protein 2;ELMO domain-containing protein 2 (Fragment),ELMO domain-containing protein 2 (Fragment),ELMO domain-containing protein 2 (Fragment)
LSS		Lanosterol synthase;Lanosterol synthase (Fragment),Lanosterol synthase (Fragment),Lanosterol synthase (Fragment),Lanosterol synthase (Fragment);Terpene cyclase/mutase family member
GALK1	HEL-S-19 ; GALK	Galactokinase,Galactokinase;Epididymis secretory protein Li 19;Galactokinase (Fragment),Galactokinase (Fragment),Galactokinase (Fragment),Galactokinase (Fragment),Galactokinase (Fragment),Galactokinase (Fragment),Galactokinase (Fragment)
GCC2		GRIP and coiled-coil domain-containing protein 2,GRIP and coiled-coil domain-containing protein 2;GRIP and coiled-coil domain-containing protein 2 (Fragment),GRIP and coiled-coil domain-containing protein 2 (Fragment),GRIP and coiled-coil domain-containing protein 2 (Fragment),GRIP and coiled-coil domain-containing protein 2 (Fragment),GRIP and coiled-coil domain-containing protein 2 (Fragment),GRIP and coiled-coil domain-containing protein 2 (Fragment);GRIP and coiled-coil domain containing 2, isoform CRA_a
ACBD3		Golgi resident protein GCP60;Acyl-Coenzyme A binding domain containing 3, isoform CRA_a
GMDS		GDP-mannose 4,6 dehydratase;cDNA, FLJ94599, highly similar to Homo sapiens GDP-mannose 4,6-dehydratase (GMDS), mRNA
GNS		N-acetylglucosamine-6-sulfatase,N-acetylglucosamine-6-sulfatase;Glucosamine (N-acetyl)-6-sulfatase (Sanfilippo disease IIID), isoform CRA_a;cDNA FLJ75883, highly similar to Homo

		sapiens glucosamine (N-acetyl)-6-sulfatase (Sanfilippo disease IIID) (GNS), mRNA
GRHPR		Glyoxylate reductase/hydroxypyruvate reductase
HSPA5	HEL-S-89n	78 kDa glucose-regulated protein;Epididymis secretory sperm binding protein Li 89n
GSR		Glutathione reductase, mitochondrial;Epididymis luminal protein 75
SLC2A14	SLC2A3	Solute carrier family 2, facilitated glucose transporter member 14,Solute carrier family 2, facilitated glucose transporter member 14;Solute carrier family 2, facilitated glucose transporter member 3,Solute carrier family 2, facilitated glucose transporter member 3;cDNA FLJ57557, highly similar to Solute carrier family 2, facilitated glucose transporter member 3;cDNA FLJ58410, highly similar to Solute carrier family 2, facilitated glucosetransporter member 3;Solute carrier family 2 (Facilitated glucose transporter), member 3 variant (Fragment);cDNA FLJ56136, highly similar to Solute carrier family 2, facilitated glucosetransporter member 14
GYS1		Glycogen [starch] synthase, muscle;Glycogen synthase 1 (Muscle) variant (Fragment);cDNA FLJ76881, highly similar to Homo sapiens glycogen synthase 1 (muscle) (GYS1), mRNA
HMMR		Hyaluronan mediated motility receptor;HMMR protein (Fragment),HMMR protein (Fragment),HMMR protein (Fragment),HMMR protein (Fragment),HMMR protein (Fragment)
HNRNPCL1	HNRNPCL3 ; HNRNPCL4	Heterogeneous nuclear ribonucleoprotein C-like 1;Heterogeneous nuclear ribonucleoprotein C-like 3;Heterogeneous nuclear ribonucleoprotein C-like 4,Heterogeneous nuclear ribonucleoprotein C-like 4;HNRPCL1 protein (Fragment)
HVCN1		Voltage-gated hydrogen channel 1,Voltage-gated hydrogen channel 1;Hydrogen voltage-gated channel 1 variant 3
IDI1		Isopentenyl-diphosphate Delta-isomerase 1
IMPA1		Inositol monophosphatase 1,Inositol monophosphatase 1,Inositol monophosphatase 1,Inositol monophosphatase 1;Inositol(Myo)-1(Or 4)-monophosphatase 1, isoform CRA_a
IMPA2		Inositol monophosphatase 2
IQGAP2		Ras GTPase-activating-like protein IQGAP2,Ras GTPase-activating-like protein IQGAP2,Ras GTPase-activating-like protein IQGAP2,Ras GTPase-activating-like protein IQGAP2;IQ motif containing GTPase activating protein 2, isoform CRA_b;Ras GTPase-activating-like protein IQGAP2 (Fragment),Ras GTPase-activating-like protein IQGAP2 (Fragment),Ras GTPase-activating-like protein IQGAP2 (Fragment),Ras GTPase-activating-like protein IQGAP2 (Fragment),Ras GTPase-activating-like protein IQGAP2 (Fragment)
ISOC2		Isochorismatase domain-containing protein 2, mitochondrial;Isochorismatase domain-containing protein 2, mitochondrial (Fragment),Isochorismatase domain-containing protein 2, mitochondrial (Fragment)
ITGA5		Integrin alpha-5,Integrin alpha-5;Integrin, alpha 5 (Fibronectin receptor, alpha polypeptide), isoform CRA_b;cDNA, FLJ92752, highly similar to Homo sapiens integrin, alpha 5 (fibronectin receptor, alphapolypeptide) (ITGA5), mRNA;cDNA FLJ77742, highly similar to Homo sapiens integrin, alpha 5 (fibronectin receptor, alpha polypeptide), mRNA
KIF1BP		KIF1-binding protein
CMPK1	CMPK	UMP-CMP kinase,UMP-CMP kinase,UMP-CMP kinase,UMP-CMP kinase;cDNA FLJ53966, moderately similar to Homo sapiens cytidylate kinase (CMPK), mRNA
PRPSAP2		Phosphoribosyl pyrophosphate synthase-associated protein 2,Phosphoribosyl pyrophosphate synthase-associated protein 2,Phosphoribosyl pyrophosphate synthase-associated protein 2,Phosphoribosyl pyrophosphate synthase-associated protein 2;Phosphoribosyl pyrophosphate synthase-associated protein 2 (Fragment),Phosphoribosyl pyrophosphate synthase-associated protein 2 (Fragment),Phosphoribosyl pyrophosphate synthase-associated protein 2 (Fragment),Phosphoribosyl pyrophosphate synthase-associated protein 2 (Fragment),Phosphoribosyl pyrophosphate synthase-associated protein 2 (Fragment),Phosphoribosyl pyrophosphate synthase-associated protein 2 (Fragment),Phosphoribosyl pyrophosphate synthase-associated protein 2 (Fragment),Phosphoribosyl pyrophosphate synthase-associated protein 2 (Fragment),Phosphoribosyl pyrophosphate synthase-associated protein 2 (Fragment),Phosphoribosyl pyrophosphate synthase-associated protein 2 (Fragment),Phosphoribosyl pyrophosphate synthase-associated protein 2 (Fragment);Phosphoribosyl pyrophosphate synthetase-associated protein 2, isoform CRA_a;cDNA, FLJ93570, highly similar to Homo sapiens phosphoribosyl pyrophosphate synthetase-associated protein 2 (PRPSAP2), mRNA
LGALS1		Galectin-1
GLO1	HEL-S-74	Lactoylglutathione lyase,Lactoylglutathione lyase;Lactoylglutathione lyase (Fragment)
LTA4H		Leukotriene A-4 hydrolase
LYZ	LYZC; LYZF1	Lysozyme C,Lysozyme C;Lysozyme,Lysozyme,Lysozyme
MAGT1		Magnesium transporter protein 1,Magnesium transporter protein 1;cDNA FLJ56344, highly

		similar to Implantation-associated protein
APOOL		MICOS complex subunit MIC27,MICOS complex subunit MIC27,MICOS complex subunit MIC27;Putative uncharacterized protein DKFZp779P1227
MLEC		Malectin,Malectin;Malectin (Fragment),Malectin (Fragment)
MPI		Mannose-6-phosphate isomerase,Mannose-6-phosphate isomerase,Mannose-6-phosphate isomerase,Mannose-6-phosphate isomerase,Mannose-6-phosphate isomerase,Mannose-6-phosphate isomerase,Mannose-6-phosphate isomerase,Mannose-6-phosphate isomerase,Mannose-6-phosphate isomerase,Mannose-6-phosphate isomerase;Mannose-6-phosphate isomerase (Fragment),Mannose-6-phosphate isomerase (Fragment),Mannose-6-phosphate isomerase (Fragment),Mannose-6-phosphate isomerase (Fragment),Mannose-6-phosphate isomerase (Fragment),Mannose-6-phosphate isomerase (Fragment),Mannose-6-phosphate isomerase (Fragment),Mannose-6-phosphate isomerase (Fragment),Mannose-6-phosphate isomerase (Fragment);cDNA FLJ56688, highly similar to Mannose-6-phosphate isomerase (EC 5.3.1.8);Mannose phosphate isomerase isoform
MYLK		Myosin light chain kinase, smooth muscle,Myosin light chain kinase, smooth muscle
PPP1R12A	PPP1R12B	Protein phosphatase 1 regulatory subunit 12A;Protein phosphatase 1 regulatory subunit,Protein phosphatase 1 regulatory subunit,Protein phosphatase 1 regulatory subunit,Protein phosphatase 1 regulatory subunit,Protein phosphatase 1 regulatory subunit,Protein phosphatase 1 regulatory subunit;Protein phosphatase 1 regulatory subunit 12A (Fragment),Protein phosphatase 1 regulatory subunit 12A (Fragment),Protein phosphatase 1 regulatory subunit 12A (Fragment),Protein phosphatase 1 regulatory subunit 12A (Fragment),Protein phosphatase 1 regulatory subunit 12A (Fragment),Protein phosphatase 1 regulatory subunit 12A (Fragment),Protein phosphatase 1 regulatory subunit 12A (Fragment),Protein phosphatase 1 regulatory subunit 12A (Fragment)
NADSYN1		Glutamine-dependent NAD(+) synthetase,Glutamine-dependent NAD(+) synthetase,Glutamine-dependent NAD(+) synthetase,Glutamine-dependent NAD(+) synthetase;Glutamine-dependent NAD(+) synthetase (Fragment),Glutamine-dependent NAD(+) synthetase (Fragment),Glutamine-dependent NAD(+) synthetase (Fragment),Glutamine-dependent NAD(+) synthetase (Fragment),Glutamine-dependent NAD(+) synthetase (Fragment)
NAF1		H/ACA ribonucleoprotein complex non-core subunit NAF1
NCAM2		Neural cell adhesion molecule 2,Neural cell adhesion molecule 2;Neural cell adhesion molecule 2, isoform CRA_a (Fragment);cDNA FLJ54289, highly similar to Neural cell adhesion molecule 2
NDRG3		Protein NDRG3,Protein NDRG3;NDRG family member 3, isoform CRA_c;cDNA FLJ54521, highly similar to Protein NDRG3;NDRG3 protein;cDNA FLJ52497, highly similar to Protein NDRG3
APOA1BP		NAD(P)H-hydrate epimerase,NAD(P)H-hydrate epimerase,NAD(P)H-hydrate epimerase;cDNA FLJ56357, highly similar to Homo sapiens apolipoprotein A-I binding protein (APOA1BP), mRNA
NOP14		Nucleolar protein 14,Nucleolar protein 14;cDNA FLJ76065
NAP1L1		Nucleosome assembly protein 1-like 1,Nucleosome assembly protein 1-like 1,Nucleosome assembly protein 1-like 1,Nucleosome assembly protein 1-like 1,Nucleosome assembly protein 1-like 1,Nucleosome assembly protein 1-like 1,Nucleosome assembly protein 1-like 1;Nucleosome assembly protein 1-like 1 (Fragment),Nucleosome assembly protein 1-like 1 (Fragment),Nucleosome assembly protein 1-like 1 (Fragment),Nucleosome assembly protein 1-like 1 (Fragment),Nucleosome assembly protein 1-like 1 (Fragment),Nucleosome assembly protein 1-like 1 (Fragment),Nucleosome assembly protein 1-like 1 (Fragment),Nucleosome assembly protein 1-like 1 (Fragment),Nucleosome assembly protein 1-like 1 (Fragment)
ORC3		Origin recognition complex subunit 3;cDNA FLJ54334, highly similar to Origin recognition complex subunit 3
SMEK1	PPP4R3A; KIAA2010	Serine/threonine-protein phosphatase 4 regulatory subunit 3A,Serine/threonine-protein phosphatase 4 regulatory subunit 3A;KIAA2010, isoform CRA_a
PAPOLA		Poly(A) polymerase alpha,Poly(A) polymerase alpha,Poly(A) polymerase alpha,Poly(A) polymerase alpha;Poly(A) polymerase alpha, isoform CRA_a;Poly(A) polymerase alpha (Fragment),Poly(A) polymerase alpha (Fragment),Poly(A) polymerase alpha (Fragment),Poly(A) polymerase alpha (Fragment),Poly(A) polymerase alpha (Fragment);cDNA FLJ60112, highly similar to Poly(A) polymerase alpha (EC 2.7.7.19);cDNA FLJ56176, highly similar to Poly(A) polymerase alpha (EC 2.7.7.19)
PCYT2		Ethanolamine-phosphate cytidylyltransferase,Ethanolamine-phosphate cytidylyltransferase,Ethanolamine-phosphate cytidylyltransferase;Ethanolamine-phosphate cytidylyltransferase (Fragment),Ethanolamine-phosphate cytidylyltransferase

		(Fragment),Ethanolamine-phosphate cytidylyltransferase (Fragment),Ethanolamine-phosphate cytidylyltransferase (Fragment),Ethanolamine-phosphate cytidylyltransferase (Fragment),Ethanolamine-phosphate cytidylyltransferase (Fragment)
PFKP		ATP-dependent 6-phosphofructokinase, platelet type,ATP-dependent 6-phosphofructokinase, platelet type
PGK1	HEL-S-68p; HEL-S-272	Phosphoglycerate kinase 1;Phosphoglycerate kinase,Phosphoglycerate kinase,Phosphoglycerate kinase,Phosphoglycerate kinase,Phosphoglycerate kinase,Phosphoglycerate kinase
PLG		Plasminogen
PLOD1		Procollagen-lysine,2-oxoglutarate 5-dioxygenase 1;Procollagen-lysine 1, 2-oxoglutarate 5-dioxygenase 1, isoform CRA_a;cDNA FLJ53377, highly similar to Procollagen-lysine, 2-oxoglutarate 5-dioxygenase 1 (EC 1.14.11.4);cDNA, FLJ92537, highly similar to Homo sapiens procollagen-lysine, 2-oxoglutarate 5-dioxygenase (lysine hydroxylase, Ehlers-Danlos syndrome type VI) (PLOD), mRNA
PRTN3		Myeloblastin
HNRPU		Heterogeneous nuclear ribonucleoprotein U (Scaffold attachment factor A), isoform CRA_a
RASSF2		Ras association domain-containing protein 2
SCPEP1		Retinoid-inducible serine carboxypeptidase,Retinoid-inducible serine carboxypeptidase,Retinoid-inducible serine carboxypeptidase;cDNA FLJ55993, highly similar to Retinoid-inducible serine carboxypeptidase (EC 3.4.16.-)
MRPL23		39S ribosomal protein L23, mitochondrial,39S ribosomal protein L23, mitochondrial,39S ribosomal protein L23, mitochondrial,39S ribosomal protein L23, mitochondrial;39S ribosomal protein L23, mitochondrial (Fragment);Mitochondrial ribosomal protein L23, isoform CRA_a;cDNA, FLJ94423, highly similar to Homo sapiens mitochondrial ribosomal protein L23 (MRPL23), nuclear gene encoding mitochondrial protein, mRNA
RPN2		Dolichyl-diphosphooligosaccharide--protein glycosyltransferase subunit 2
DAP3		28S ribosomal protein S29, mitochondrial,28S ribosomal protein S29, mitochondrial
S100A4		Protein S100-A4
SCFD1		Sec1 family domain-containing protein 1,Sec1 family domain-containing protein 1,Sec1 family domain-containing protein 1,Sec1 family domain-containing protein 1,Sec1 family domain-containing protein 1,Sec1 family domain-containing protein 1,Sec1 family domain-containing protein 1;Vesicle transport-related protein isoform a variant (Fragment)
BNIP1		Vesicle transport protein SEC20
SH3KBP1		SH3 domain-containing kinase-binding protein 1,SH3 domain-containing kinase-binding protein 1;GIG10
SH3GLB1		Endophilin-B1
NANS		Sialic acid synthase
SLK		STE20-like serine/threonine-protein kinase
SEPHS1		Selenide, water dikinase 1
STX6		Syntaxin-6
STX7		Syntaxin-7
SYNE1		Nesprin-1
TUBG1		Tubulin gamma-1 chain
TTC4		Tetratricopeptide repeat protein 4
TTC5		Tetratricopeptide repeat protein 5
TMSB4X		Thymosin beta-4;Thymosin beta 4, X-linked;TMSB4X protein (Fragment),TMSB4X protein (Fragment),TMSB4X protein (Fragment),TMSB4X protein (Fragment),TMSB4X protein (Fragment)
HEL-S-26		Isocitrate dehydrogenase [NADP]
KIAA1033		WASH complex subunit 7,WASH complex subunit 7,WASH complex subunit 7,WASH complex subunit 7;KIAA1033 protein;WASH complex subunit 7 (Fragment),WASH complex subunit 7 (Fragment),WASH complex subunit 7 (Fragment)
WBP2		WW domain-binding protein 2,WW domain-binding protein 2,WW domain-binding protein 2,WW domain-binding protein 2,WW domain-binding protein 2,WW domain-binding protein 2;WW domain binding protein 2, isoform CRA_a;WW domain-binding protein 2 (Fragment),WW domain-binding protein 2 (Fragment),WW domain-binding protein 2 (Fragment),WW domain-binding protein 2 (Fragment);WW-domain binding protein 2
XPO4		Exportin-4,Exportin-4;Exportin 4
ZNF622		Zinc finger protein 622
ZNF841		Zinc finger protein 841;Zinc finger protein 841 (Fragment),Zinc finger protein 841 (Fragment)

Supplementary Table S2. List of the m/z recorded for each isotopomer of different fatty acids studied by GC-MS.

	C14:0	C16:1	C16:0	C18:1	C18:0	C20:4
**Retention Time (min)**	12.29	14.16	14.36	16.06	16.28	17.39
**M0**	227,3	253,3	255,3	281,3	283,3	303,3
**M+1**	228,3	254,3	256,3	282,3	284,3	304,3
**M+2**	229,3	255,3	257,3	283,3	285,3	305,3
**M+3**	230,3	256,3	258,3	284,3	286,3	306,3
**M+4**	231,3	257,3	259,3	285,3	287,3	307,3
**M+5**	232,3	258,3	260,3	286,3	288,3	308,3
**M+6**	233,3	259,3	261,3	287,3	289,3	309,3
**M+7**	234,3	260,3	262,3	288,3	290,3	310,3
**M+8**	235,3	261,3	263,3	289,3	291,3	311,3
**M+9**	236,3	262,3	264,3	290,3	292,3	312,3
**M+10**	237,3	263,3	265,3	291,3	293,3	313,3
**M+11**	238,3	264,3	266,3	292,3	294,3	314,3
**M+12**	239,3	265,3	267,3	293,3	295,3	315,3
**M+13**	240,3	266,3	268,3	294,3	296,3	316,3
**M+14**	241,3	267,3	269,3	295,3	297,3	317,3
**M+15**		268,3	270,3	296,3	298,3	318,3
**M+16**		269,3	271,3	297,3	299,3	319,3
**M+17**				298,3	300,3	320,3
**M+18**				299,3	301,3	321,3
**M+19**						322,3
**M+20**						323,3

Supplementary Figure S1. Detail of relative quantification of molecular species obtained by LC-MS for PI (A) an for ceramides (B).

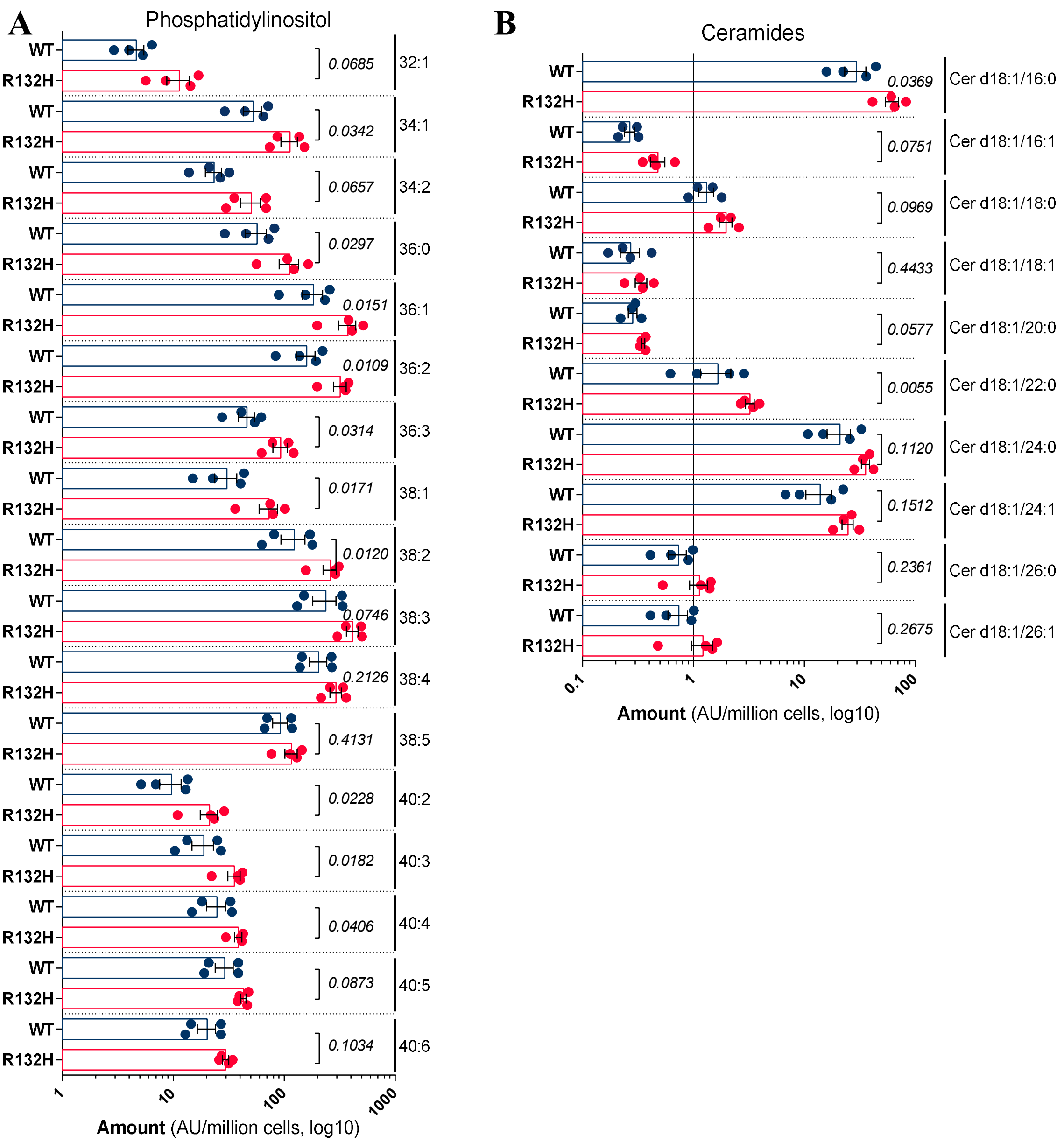

Supplement: Supplementary file 1 [file ijms-19-03325-s001.pdf]
